# A framework for testing assumptions about foraging scales, body mass, and niche separation using telemetry data

**DOI:** 10.1002/ece3.3078

**Published:** 2017-06-08

**Authors:** Graeme S. Cumming, Dominic A.W. Henry, Chevonne Reynolds

**Affiliations:** ^1^ Percy FitzPatrick Institute DST/NRF Centre of Excellence University of Cape Town Rondebosch Cape Town South Africa; ^2^ ARC Centre of Excellence for Coral Reef Studies James Cook University Townsville QLD Australia; ^3^ Statistics in Ecology, Environment and Conservation Department of Statistical Sciences University of Cape Town Rondebosch Cape Town South Africa

**Keywords:** body size, dispersal, hierarchy, movement, multimodality, scale, scaling, southern Africa

## Abstract

Ecological theory predicts that if animals with very similar dietary requirements inhabit the same landscape, then they should avoid niche overlap by either exploiting food resources at different times or foraging at different spatial scales. Similarly, it is often assumed that animals that fall in different body mass modes and share the same body plan will use landscapes at different spatial scales. We developed a new methodological framework for understanding the scaling of foraging (i.e. the range and distribution of scales at which animals use their landscapes) by applying a combination of three well‐established methods to satellite telemetry data to quantify foraging patch size distributions: (1) first‐passage time analysis; (2) a movement‐based kernel density estimator; and (3) statistical comparison of resulting histograms and tests for multimodality. We demonstrate our approach using two sympatric, ecologically similar species of African ducks with quite different body masses: Egyptian Geese (actually a shelduck), and Red‐billed Teal. Contrary to theoretical predictions, the two species, which are sympatric throughout the year, foraged at almost identical spatial scales. Our results show how ecologists can use GPS tracking data to explicitly quantify and compare the scales of foraging by different organisms within an animal community. Our analysis demonstrates both a novel approach to foraging data analysis and the need for caution when making assumptions about the relationships among niche separation, diet, and foraging scale.

## 1 INTRODUCTION

One of the fundamental questions of community ecology is that of what drives variation in the numbers and kinds of species that occur in different ecosystems. Available energy, resources, habitat structure, and interspecific interactions have long been known to be important influences on community composition (Clements, 1916; Gleason, 1926; Elton, 1927). However, different perspectives on ecological structure and organization (e.g. food web ecology, ecosystem ecology, landscape ecology, and population ecology) are still poorly integrated. One of the reasons for this lack of integration is that incorporation of spatial heterogeneity and animal movement ecology into each of these bodies of theory has been difficult and has proceeded in different ways in different fields. For example, Elton's (1927) trophic pyramid provides a first‐principles explanation for why predator biomass will always be lower than that of herbivores, but newer understandings of the importance of spatial heterogeneity for herbivory and predation (e.g. Hebblewhite & Merrill, 2009; Pascual, Mazzega, & Levin, 2001; Ripple & Beschta, 2003) have not been translated into corresponding predictions about spatial variance in biomass.

Despite making progress in such areas as the study of metapopulations, metacommunities, and Allee effects, ecologists have not yet fully reconciled niche theory (and related ideas about fundamental ecological mechanisms that occur at the level of individuals, such as foraging, competition, and predation) with landscape and community ecology. While we have the beginnings of a “first‐principles theory” with roots in body mass, body plan (e.g. legs, wings, or fins), and metabolic rate (Brown, 1984, 1995), these mechanisms have proved difficult to translate into predictions about broader‐scale patterns in community composition (Ritchie & Olff, 1999).

Body mass and plan can usefully explain and predict many key elements of an animal's ecology, including such things as its metabolic rate, resource demands, locomotory performance, life expectancy, and reproductive rate (Schmidt‐Nielsen, 1984; Dees, Hofmann, & Bahar, 2010). Animals must solve the problem of reproducing in a variable environment in which they must both find resources and successfully interact (both positively and negatively, through activities such as reproduction and predation) with other animals (Kooijman & Lika, 2014). Having a larger body mass has ecological implications that include benefits (e.g. improved thermoregulatory ability, escaping predation by smaller carnivores, or being able to eat a greater range of prey) and costs (e.g. requiring more resources, being more conspicuous, and adapting to environmental change more slowly because of a slower reproductive rate) (Peters, 1983).

Body mass also has a strong evolutionary component (Sibly & Brown, 2007). Classical theories about the processes that drive the evolution of body mass assigned primacy to interspecific interactions (Hutchinson, 1959). Of particular relevance to the methods presented in this paper is the proposal by Holling (1992) that patterns in body mass distributions within a community are produced by unevenness (clustering) in the cross‐scale distribution of resources. His “Textural Discontinuity Hypothesis” (TDH) proposes that some body masses (and presumably, body plans) are untenable in some landscapes because of either a lack of resources that would support an organism of that body plan and size or superiority in competitive ability at some scales (Szabo & Meszena, 2006; Fischer, Lindenmayer, & Montague‐Drake, 2008); while other body masses are commoner than might be expected, they allow organisms to exploit rich pockets of resources that exist at particular scales within the landscape (Holling, 1992).

Contemporary ecological hypotheses that seek to explain body mass distributions (such as the TDH) treat body mass as both a driver and a response variable, making it difficult to distinguish between the causes (in evolutionary time) and the consequences (in ecological time) of body mass. Most empirical analyses of such hypotheses have not yet gone beyond the use of body mass distributions as the primary data source (Allen et al., 2006). As a result, our understanding of body mass and body plan as influences on the ecology and evolution of organisms has been only weakly connected to the landscape ecology of animal movement and foraging. Surprisingly, few empirical tests of assumptions about the scaling of animal foraging patterns in relation to body size have been undertaken.

While there is already considerable support for the idea that most vertebrates focus their foraging efforts on resource‐rich patches, with longer “commuting” movements between foraging patches (Fauchald & Tveraa, 2006; de Knegt, Hengeveld, van Langevelde, de Boer, & Kirkman, 2007), these analyses do not usually address the question of how animal foraging movements are distributed *across different scales*. That is, they do not directly assess the assumptions made by the TDH and other theories about the scaling relationships between body mass and resource distributions. Specifically, there is a clear need for more rigorous tests of the widely held assumptions that (1) foraging effort within a community of sympatric animals occurs across multiple scales, rather than being centered around a single scale; and (2) there are scale breaks (i.e., potentially available intermediate scales of foraging activity that are not apparent in data) in the spatial extents of foraging movements.

One of the reasons why telemetry data have not been used to test community‐level hypotheses about foraging and body size is presumably that appropriate frameworks for doing so have not been established. However, telemetry data have the potential to let the animals themselves tell us where food is in their environment and how they use it, rather than attempting to measure food distributions ourselves. In this paper, we address this methodological gap, presenting a novel approach to the quantitative, empirical analysis of scale and foraging behavior. We demonstrate our approach using a combination of dietary information and GPS satellite telemetry data from two African ducks: Red‐billed Teal (*Anas erythrorhyncha*) and Egyptian Geese (*Alopochen aegyptiaca*).

Despite their many morphological, phylogenetic, and ecological similarities, Red‐billed Teal and Egyptian Geese tracked in this study weighed 642± SD 97 g and 2299± SD 43 g, respectively (Cumming & Ndlovu, 2011). Egyptian Geese are thus approximately three and a half times larger than Red‐billed Teal. Our prediction, given the similarities in their diets and the differences in their body masses, was that we would find evidence for niche separation in the form of clear‐cut differences in the scales at which these species forage across the landscape. Note that in this analysis, we do not attempt to directly test the TDH or related explanations; we simply seek to develop and demonstrate the virtues of a method by which more exhaustive studies of animal communities, using a much larger range of species, could approach the problem.

## 2 METHODS

Our approach combines telemetry data, first‐passage time analysis, home‐range analysis, comparison of frequency histograms, and finally a test for multimodality. We will go through each of these steps in detail.

### 2.1 Telemetry data, study sites, and species

**Figure 1:**
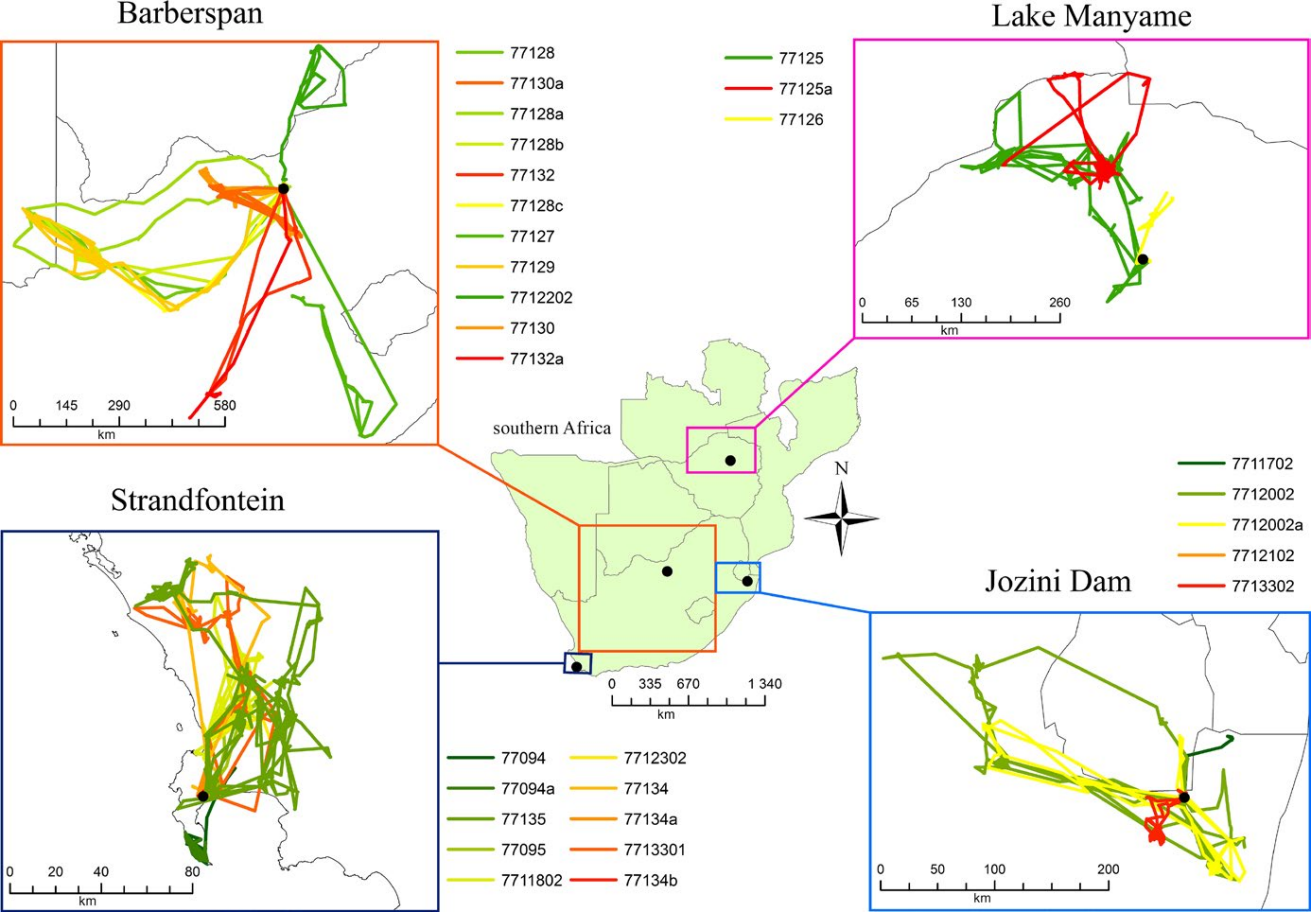
Overview of southern Africa, showing the locations and tracks of all Egyptian Geese included in the study

**Figure 2:**
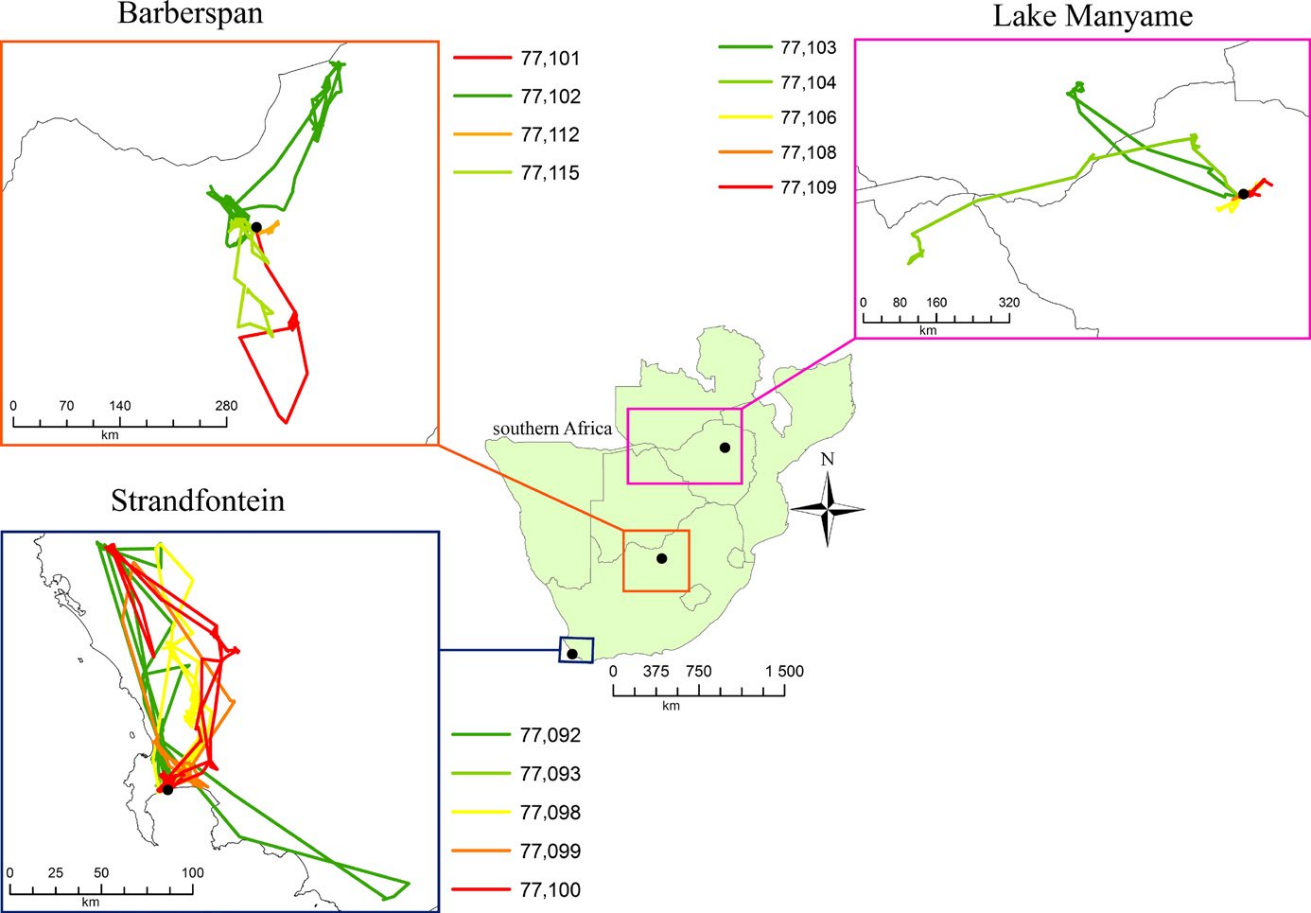
Overview of southern Africa, showing the locations and tracks of all Red‐billed Teal included in the study

The test data set included movement paths from 19 Egyptian Geese and 14 Red‐billed Teal that were tagged at four quite different wetlands (Figures 1 and 2): Strandfontein (Western Cape, South Africa, with winter rainfall), Barberspan (Northwest province, South Africa, with highly variable and generally low rainfall), Lake Manyame (near Harare, Zimbabwe, with summer rainfall), and Jozini Dam (northern KwaZulu‐Natal, South Africa, near the Mozambique and Swaziland borders, with seasonally variable but relatively high summer rainfall). Birds were monitored during the period 2008–2013 for periods of time ranging from 111 days (c. 3.5 months) to 1126 days (c. 3 years; see Table 1).

**Table 1:** Summary of each individual included in the analysis, giving their satellite GPS PTT number, tagging site, total number of relocations obtained, start date, days tracked, total distance moved (km), and mean distance per day

Site	Spp	PTT	Number of relocations	Start date	End date	Days	Dist (km)	km day^−1^
BAR	EG	7712202	2123	10/23/2008	5/30/2009	219	2491.18	11.38
BAR	EG	77127	6351	6/7/2008	5/10/2010	702	5587.85	7.96
BAR	EG	77128	3551	6/22/2008	6/6/2009	349	3041.57	8.72
BAR	EG	77128a	2551	8/15/2009	5/25/2010	283	3613.02	12.77
BAR	EG	77128b	1998	9/25/2010	5/6/2011	223	2882.35	12.93
BAR	EG	77128c	653	7/31/2011	12/2/2011	124	1159.43	9.35
BAR	EG	77129	3491	6/7/2008	5/15/2009	342	5265.88	15.40
BAR	EG	77130	2489	11/9/2008	9/19/2009	314	4645.91	14.80
BAR	EG	77130a	2140	10/4/2009	6/4/2010	243	3420.21	14.07
BAR	EG	77132	2601	6/7/2008	5/30/2009	357	3029.3	8.49
BAR	EG	77132a	2090	8/13/2009	4/14/2010	244	1744.4	7.15
BAR	RBT	77101	740	4/9/2008	9/28/2008	172	887.33	5.16
BAR	RBT	77102	4155	4/10/2008	4/20/2010	740	4161.22	5.62
BAR	RBT	77112	1843	6/7/2008	5/15/2009	342	1655.75	4.84
BAR	RBT	77115	1429	10/11/2008	7/15/2009	277	1616.16	5.83
JOZ	EG	7711702	1669	5/4/2012	9/20/2012	139	345.92	2.49
JOZ	EG	7712002	4317	5/4/2012	5/24/2013	385	5753.11	14.94
JOZ	EG	7712002a	2592	6/9/2013	1/31/2014	236	2806.24	11.89
JOZ	EG	7712102	1309	5/5/2012	9/3/2012	121	118.61	0.98
JOZ	EG	7713302	3009	5/4/2012	2/19/2013	291	975.21	3.35
MAN	EG	77125	6965	5/7/2008	2/21/2010	655	10997.2	16.79
MAN	EG	77125a	3689	4/17/2010	5/31/2011	409	5189.95	12.69
MAN	EG	77126	2682	5/7/2008	12/26/2008	233	2356.35	10.11
MAN	RBT	77103	610	5/5/2008	8/24/2008	111	1651.6	14.88
MAN	RBT	77104	1431	5/5/2008	1/25/2009	265	2366.17	8.93
MAN	RBT	77106	2587	5/6/2008	7/25/2009	445	2149.57	4.83
MAN	RBT	77108	644	5/6/2008	8/29/2008	115	711.25	6.18
MAN	RBT	77109	1307	5/7/2008	12/24/2008	231	1253.39	5.43
STR	EG	77094	1218	1/12/2008	5/9/2008	118	537.2	4.55
STR	EG	77094a	2686	8/20/2008	5/1/2009	254	1038.44	4.09
STR	EG	77095	3397	1/12/2008	1/3/2009	357	1798.93	5.04
STR	EG	7711802	6453	1/17/2009	10/11/2010	632	4201.96	6.65
STR	EG	7712302	1756	12/5/2008	6/2/2009	179	543.54	3.04
STR	EG	7713301	1506	12/4/2008	4/27/2009	144	1082.52	7.52
STR	EG	77134	5330	12/1/2008	7/29/2010	605	1893.01	3.13
STR	EG	77134a	2561	8/19/2010	5/2/2011	256	1144.43	4.47
STR	EG	77134b	2401	7/22/2011	4/12/2012	265	881.83	3.33
STR	EG	77135	8522	12/1/2008	2/8/2011	799	5155.63	6.45
STR	RBT	77092	1804	3/12/2008	3/26/2009	379	2217.02	5.85
STR	RBT	77093	993	3/12/2008	9/7/2008	179	611.1	3.41
STR	RBT	77098	3550	3/14/2008	11/24/2009	620	2173.4	3.51
STR	RBT	77099	1859	3/14/2008	5/15/2009	427	1532.42	3.59
STR	RBT	77100	2046	3/14/2008	4/16/2009	398	1445.63	3.63

RBT, Red‐billed Teal; EG, Egyptian Goose. Sites are (as displayed in Figure 1): STR, Strandfontein; BAR, Barberspan; MAN, Manyame; JOZ, Jozini.

All ducks were equipped with platform transmitter terminals (PTTs). We used 22‐g PTTs for Red‐billed Teal (RBT) and 32‐g PTTs for Egyptian Geese (EG). Note that Egyptian Geese are ducks in the family Tadorninae (shelducks), rather than true geese. PTTs were set to capture GPS location data every 2 hr (EG) and 4 hr (RBT) (Cumming, Gaidet, & Ndlovu, 2012). The location data are high resolution (accuracy c. 10–20 m), and the study species are highly mobile, making smaller errors in location (e.g. at the scale of 50–100 m) both difficult to detect and irrelevant to our conclusions. The duration between fixes in the tracks of each bird was inspected, and tracks were split if the time between fixes was greater than 1 week (split tracks of each individual are denoted as either a, b, or c dependent on the number of sampling gaps detected – see Table 1).

An understanding of the ecology of our study species is important for interpreting our data. Egyptian Geese and Red‐billed Teal are ubiquitous in southern Africa. Egyptian Geese are typically classified as grazing ducks that forage primarily on land, while Red‐billed Teal are considered to be dabbling ducks that forage primarily in water (Hockey, Dean, & Ryan, 2005). However, both species are commonly found at wetlands, and both may range far from open water to forage on agricultural fields. Both species also exhibit a relatively predictable daily movement sequence in most locations, with birds roosting overnight and foraging intensively in the morning and evening (Hockey et al., 2005; Ndlovu, Cumming, & Hockey, 2014). Their movements throughout the year can be divided into three main phases: (1) breeding, during which they are tied to a nest site and a nearby wetland until the ducklings can fly (duration around 10 to 15 weeks in total, with 4–5 weeks of incubation and 8–10 weeks for the ducklings to grow); (2) flightless molt, which involves synchronous replacement of the primaries at a deep, permanent wetland and takes 4–5 weeks every year (Milstein, 1993); and (3) a “roaming” period, during which the birds move around the landscape in flocks, following food resources in a seminomadic manner. Full analyses of the movements of both species have already been published (Cumming et al., 2012; Ndlovu, Cumming, Hockey, Nkosi, & Mutumi, 2013; Ndlovu et al., 2014).

Our study species show strong dietary overlap. Red‐billed Teal are typically categorized as omnivorous dabbling ducks and Egyptian Goose as herbivorous grazing ducks, but these categorizations are not well supported by recent evidence. A quantitative meta‐analysis of plant families in the diets of all 16 indigenous southern African waterfowl showed that Red‐billed Teal and Egyptian Goose were the most similar species in terms of their dietary composition (Reynolds & Cumming, 2016a). This similarity was largely driven by the presence of grasses (Poaceae) and agricultural grains, as well as their preferences for foraging in both terrestrial and aquatic habitats. In addition, a comparison of the composition of seed species found in the diet of Red‐billed Teal and Egyptian Goose at Barberspan showed no significant differences (Reynolds & Cumming, 2016b). Red‐billed Teal spend a significant proportion of time on land, foraging in agricultural fields and dry grassland on spilled maize, wheat, sunflower seeds, and grass seeds and are often seen alongside Egyptian Geese (Skead, 1981; Hockey et al., 2005). A study on the Nyl River floodplain, South Africa, found that 73–97% of dry mass in the upper digestive tract of adult Red‐billed Teal was land grass, *Panicum schinzii* (Petrie, 1996), suggesting that plant matter is more prevalent in the diets of Red‐billed Teal than animal matter. Conversely, Egyptian Geese have been shown to dabble and probe in shallow water, and they also eat the seeds of wetland plants from the Potamogetonaceae and Polygonaceae plant families, which are common in the diets of Red‐billed Teal (Halse, 1984; Petrie, 1996; Reynolds & Cumming, 2015, 2016a).

A proportion of Red‐billed Teal diet is composed of aquatic invertebrates, which may seasonally comprise an important food resource. Higher proportions of animal matter have been recorded in the diets of egg‐laying female teal and molting teal, and additional nutrients (protein and calcium) may be important for certain life history stages (Mitchell, 1983; Petrie, 1996). Egyptian Geese may also rely seasonally on aquatic invertebrates. For example, the fecal matter of molting geese at Voëlvlei dam, South Africa, contained almost exclusively invertebrate remains. Additionally, the eggs of resting‐phase Bryozoa and Daphnia were found in fecal matter from Egyptian Geese at locations in two separate provinces in South Africa (Reynolds & Cumming, 2015). Although aquatic invertebrates have only recently been recorded in the diet of Egyptian Goose, terrestrial invertebrates (termites, ants, beetles, and earthworms) are known to be eaten on occasion and are fairly common in the diet of chicks (Douthwaite, 1978; Milstein, 1993).

### 2.2 Analysis of telemetry data

We used first‐passage time (FPT; Fauchald & Tveraa, 2003) analysis in combination with movement‐based kernel density estimators (MKDE; Benhamou & Cornelis, 2010; Benhamou & Riotte‐Lambert, 2012) to define and measure the area of spatial clusters of relocations. FPT is used to detect areas in which animal search effort is concentrated. It is calculated at each GPS fix along a movement path as the time taken to cross a circle of a given radius. The process is repeated over a range of circles with differing radii. The peaks in variance of log‐transformed FPT at a specific radius indicate the scale at which an animal's movements are clustered. FPT has been shown to increase with circle size, and thus, in order to standardize the subsequent analysis across individuals, we calculated and applied a common population‐level radius for each species. For each species, we used the radius at which mean variance of log FPT showed a peak. FPT was calculated along an individual's path with a given radius *r*, ranging from 100 to 10,000 m at 80‐m intervals, centered on consecutive locations. The radius *r*_*max*_ is the radius at which the variance of log‐transformed FPT *var*_*fpt*_ is maximized for each individual. The mean of variance *var*_*fpt mean*_ was calculated by averaging *var*_*fpt*_ of each bird at each radius. The peak in this mean *var*_*fpt mean*_ was then taken at a population average and used as the common spatial scale for all subsequent analysis. After plotting *var*_*fpt mean*_ against radius, peaks for Egyptian Geese and Red‐billed Teal were identified as 2,180 m and 2,420 m, respectively (additional details are provided in Henry, Ament, & Cumming, 2016). This result provides a first indication of similarity in the scales at which the two species use the landscape.

Once FPT analysis was applied to each individual movement path, plots were created of GPS fixes against FPT. Lavielle's segmentation method (Lavielle, 2005) was then used to identify homogenous movement bouts within an individual's movement path using the *lavielle* function in the *adehabitatLT* R package (Calenge, 2006). The method detects break points in the movement path by minimizing a penalized contrast function (Lavielle, 2005). Given that a movement path is made up of *K* segments, the method searches for an optimal number of segments *K*_*opt*_ with which to partition the movement path. There should be a clear break in the decrease in the contrast function after *K*_*opt*_, which we identified in two ways. First, we examined the contrast function plot to visually identify the value of *K*_*opt*_ that was associated with a breakpoint in the function. The numerical output of the Lavielle function was then used to identify the last value of *K*_*opt*_ for which the second derivative of the standardized contrast function was greater than *S*. The value of *S* was set at 0.75 following recommendations of Lavielle (1999). The numerical output was used to confirm our initial choice of *K*_*opt*_. It has been suggested that *S* is sensitive to optimization when the length of the time series is <500, but none of the movement paths of the birds had fewer than 500 relocations and the graphical and numerical results agreed closely. The calculation requires two additional parameters: *K*_*max*_, 3–4 times the maximum number of segments expected in the movement path, and *L*_*min*_, the minimum number of GPS fixes required to build a segment. *K*_*max*_ was visually assessed for each individual's movement path, while *L*_*min*_ was fixed at 12 and 6 for Egyptian Geese and Red‐billed Teal, respectively, which corresponds to a minimum length of a segment equal to 24 hr.

**Figure 3:**
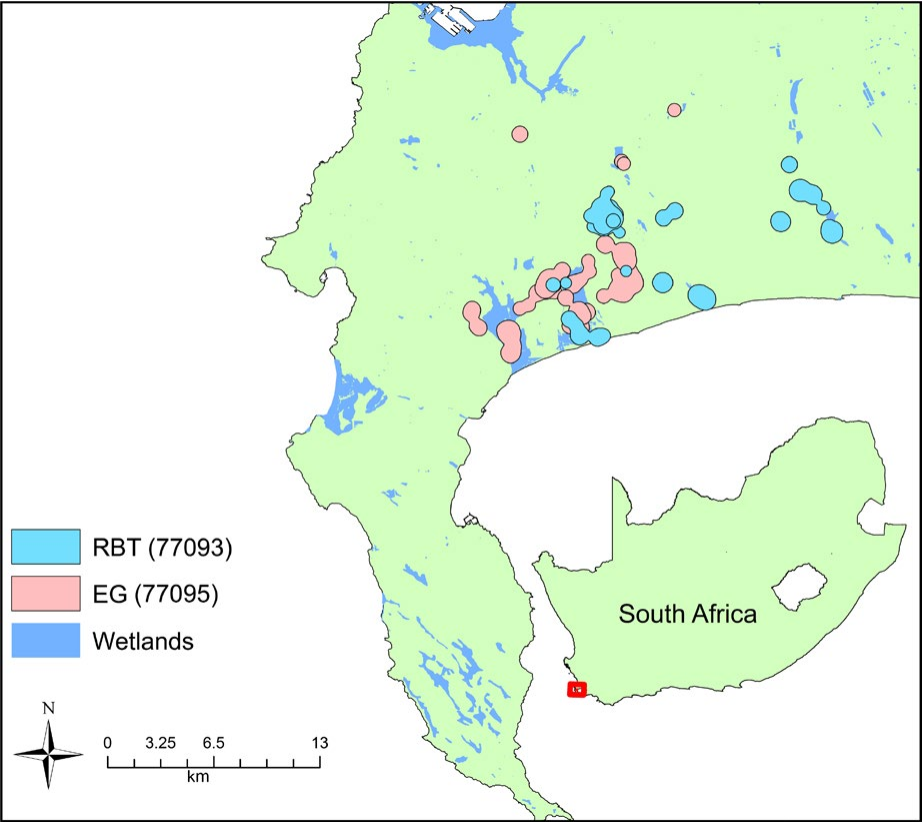
An example of first‐passage time data set showing foraging patch extents identified in the Western Cape of South Africa for two different birds, a Red‐billed Teal (RBT PTT#77093, light blue) and an Egyptian Goose (EG PTT#77095, light pink). Wetlands are shaded in darker blue

Segments from each movement path were extracted, and GPS fixes within segments were used to create a utilization distribution that defined the area of the foraging patch. The 95% utilization distributions were calculated using MKDE methods with the *BRB* function in the *adehabitatHR* R package (Calenge, 2006). MKDE is statistically superior to other approaches for these data (Cumming & Cornélis, 2012), presumably because it assigns a higher probability of use to unsampled locations that fall between known fixes, thus producing a more parsimonious model. Multiple polygons were derived for each utilization distribution as is common when using MKDE methods (Figure 3). The area of each polygon was then measured and subsequently used as a sampling unit.

### 2.3 Comparing foraging area extents and testing for multimodality

Once the extents of individual foraging areas were calculated, we visually compared the data for the two species using a smoothed density histogram. Data for both species were lognormally distributed, and we log‐transformed the data for analysis. We tested for a difference between the two histograms using the two‐sample *ks.test* function in R to run a Kolmogorov–Smirnov test for equivalence of the two distributions.

To test for multimodality within individual data sets, we ran Silverman's test (Silverman, 1981) iteratively on each foraging area data set, as implemented in the R library “*Silvermantest*” (Schwaiger & Holzmann, 2013), searching for between 1 and 30 modes. Silverman's test has been shown to be a reliable but relatively conservative test for multimodality (Xu, Bedrick, Hanson, & Restrepo, 2014).

### 2.4 Sensitivity analysis

We tested the sensitivity of our methods in two different ways. First, for each of the two different species, we sequentially added a small constant (0.01) to the foraging area data (creating a mean‐shifted data set) and tested whether it differed from the original data. Second, we pooled the original data set with each mean‐shifted data set in turn and tested whether Silverman's test could detect bimodality.

## 3 RESULTS

**Figure 4:**
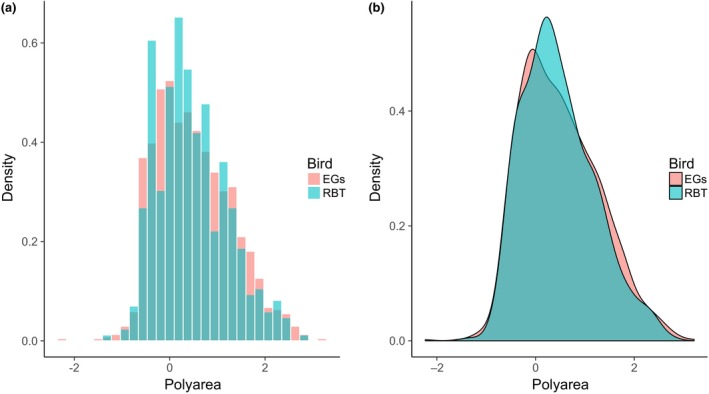
(a) Unsmoothed and (b) smoothed density histograms comparing foraging extent data for Red‐billed Teal (“rbt”, turquoise color) and Egyptian Goose (“egs”, pink color). “Polyarea” is polygon area, “density” is the proportion of points of that area

The Red‐billed Teal data yielded 457 different foraging extent polygons and the Egyptian Goose data 1,269 polygons. The density distributions of log‐transformed foraging area extents for the two species showed almost complete overlap (Figure 4). These curves were not significantly different from one another (Kolmogorov–Smirnov *D* = 0.5, *p* = .37; *n* = 457 and 1,269 for Red‐billed Teal and Egyptian Goose, respectively).

Silverman's test for multimodality, run from 1 to 30 modes, gave the highest probability of multimodality for Egyptian Geese as *p* = .6 (24 modes) and for Red‐billed Teal as 0.7 (six modes). These values are well below the recommended *p* ≥ .95 cut‐off value for accepting a hypothesis of multimodality.

Our comparisons of real data for each data set to an identical data set with a small constant added indicated that our methods can detect differences as small as 0.06 between the mean log areas of the actual versus mean‐shifted home‐range polygons for Egyptian Geese and 0.07 for the smaller data set, Red‐Billed Teal. Combining the actual Egyptian Goose data set and a mean‐shifted version of the same data similarly indicated that Silverman's test would successfully identify bimodality beyond a difference in the mean log areas of 0.07.

## 4 DISCUSSION

Although FPT, kernel density home‐range estimation, statistical comparison of frequency distributions, and tests for multimodality are all well‐established and widely used techniques, they have not previously been applied together in this context. What is particularly novel about our approach is the idea of defining foraging patches from telemetry data, rather than trying to quantify food abundance itself, and using these data to compare how different animals use and respond to the same spatial patterns of resource distributions in their landscape.

Our sensitivity analyses suggest that the methods are capable of distinguishing quite small differences between and within species. Silverman's test is known to be a conservative test for modality, but could easily replaced by a less conservative test when applying the framework; the current difficulty with alternatives, such as BCART (Bayesian Classification and Regression tree analysis), is that no alternative test has been proven to provide a fair balance between type I and type II errors. Similarly, the number of individuals from which telemetry data would be needed to provide a fair representation of population‐level trends in foraging areas (and the minimum number of fixes per individual) is unclear and needs further testing.

The unexpected outcome of our analysis demonstrates the potential value of our proposed methods. Contrary to what textural discontinuity theory would predict, we found that our two study species foraged at identical scales, or more precisely, that the frequency distributions (scales) of extents of foraging bouts were indistinguishable. Detailed analysis of their movement trajectories suggests strong similarities (this study and Cumming et al., 2012), and both species respond in similar ways to rainfall and primary production (Henry et al., 2016). The analysis therefore yields some interesting conclusions that question the validity of commonly held assumptions about optimal foraging, niche separation, and scale.

The reasons why our study species do not differentiate their foraging scales are beyond the scope of this paper. The primary messages emerging from this analysis in relation to our proposed methodology are that (1) our methods permit clear, direct comparison of patterns in foraging behavior that span multiple different scales; and (2) the methods are capable of identifying similarities and differences that may challenge existing theories and assumptions. Although we have presented an ensemble of existing methods rather than a new method per se, our overall approach has the potential to make novel contributions to the further development of theory.

The assumption that organisms that fall into different body mass modes use landscapes at different scales is seldom questioned in landscape and community ecology. While we have not yet analyzed a data set for an entire ecological community, our methods suggest that comparisons of the scales of foraging using the methods that we have presented can provide valuable tests of assumptions about niche separation and its relationship to the landscape ecology of the study group. Multiscale analysis of the foraging patterns of different individuals and species carries the intriguing potential to provide a “missing link” between individual movement data, community‐level data, and species range data (Cumming, 2007; Cumming et al., 2012). We look forward to the day when this analysis can be run across an entire community to test whether, and how, body mass patterns and foraging scales relate to one another.

## CONFLICT OF INTEREST

None declared.
